# Gamma radiation induces locus specific changes to histone modification enrichment in zebrafish and Atlantic salmon

**DOI:** 10.1371/journal.pone.0212123

**Published:** 2019-02-13

**Authors:** Leif Christopher Lindeman, Jorke Harmen Kamstra, Jarle Ballangby, Selma Hurem, Leonardo Martín Martín, Dag Anders Brede, Hans Christian Teien, Deborah H. Oughton, Brit Salbu, Jan Ludvig Lyche, Peter Aleström

**Affiliations:** 1 Centre for Environmental Radioactivity (CERAD CoE), Norwegian University of Life Sciences, Ås, Norway; 2 Faculty of Environmental Sciences and Natural Resource Management, Norwegian University of Life Sciences, Ås, Norway; 3 Faculty of Veterinary Medicine, Norwegian University of Life Sciences, Oslo, Norway; 4 Faculty of Agropecuary Sciences, University of Camagüey (UC) Ignacio Agramonte Loynaz, Camagüey, Cuba; Northwestern University Feinberg School of Medicine, UNITED STATES

## Abstract

Ionizing radiation is a recognized genotoxic agent, however, little is known about the role of the functional form of DNA in these processes. Post translational modifications on histone proteins control the organization of chromatin and hence control transcriptional responses that ultimately affect the phenotype. The purpose of this study was to investigate effects on chromatin caused by ionizing radiation in fish. Direct exposure of zebrafish *(Danio rerio)* embryos to gamma radiation (10.9 mGy/h for 3h) induced hyper-enrichment of H3K4me3 at the genes *hnf4a*, *gmnn* and *vegfab*. A similar relative hyper-enrichment was seen at the *hnf4a loci of irradiated* Atlantic salmon *(Salmo salar)* embryos (30 mGy/h for 10 days). At the selected genes in ovaries of adult zebrafish irradiated during gametogenesis (8.7 and 53 mGy/h for 27 days), a reduced enrichment of H3K4me3 was observed, which was correlated with reduced levels of histone H3 was observed. F1 embryos of the exposed parents showed hyper-methylation of H3K4me3, H3K9me3 and H3K27me3 on the same three loci, while these differences were almost negligible in F2 embryos. Our results from three selected loci suggest that ionizing radiation can affect chromatin structure and organization, and that these changes can be detected in F1 offspring, but not in subsequent generations.

## Introduction

All organisms are exposed to background levels of ionizing radiation originating from naturally occurring radionuclides and cosmic radiation. In addition, ionizing radiation can be emitted from anthropogenic sources, such as wastes from nuclear power plant facilities and medical treatment, and in extreme cases, as a result of nuclear weapons and power plant disasters. Ionizing radiation exerts its adverse effects through the formation of reactive oxygen species (ROS), reactive nitrogen species (RNS), radiation induced DNA-protein cross-links [1] and other damage to DNA, RNA and proteins [2–5]. Furthermore, ionizing radiation affects gene expression in a dose and dose rate dependent manner [6, 7], which is most likely accompanied by structural changes to chromatin. Chromatin is the functional form of the stored genetic information of the genes, allowing gene regulation control through epigenetic mechanisms.

Epigenetics can be described as mitotically and meiotically heritable changes in gene expression without changes in the DNA sequence [8]. Epigenetic mechanisms control gene expression by making the genes available or unavailable for the transcriptional machinery and can be grouped into covalent DNA modifications, post-translational modifications (PTMs) of histone proteins and expression of non-coding RNAs [9]. Covalent PTM of histones as acetylation, phosphorylation and methylation facilitate a change between transcriptionally permissive (eu-) and repressive (hetero-) chromatin states [10]. One of the most widely characterized histone PTMs is trimethylation of the lysine at the fourth position of the protruding N-terminal tail of histone H3 (H3K4me3), associated with promoters of actively transcribed genes [11, 12]. In contrast, the heterochromatin marks (H3K27me3 [13] and H3K9me3 [14–16]) are associated with repressed genes. Chromatin is known to respond to radiation induced stress (reviewed in [17]) and histone PTMs are one of many molecular candidates suggested as biomarkers for ionizing radiation [18]. However, the accumulated scientific data is not yet sufficient to enable the prediction or interpretation of the histone PTM response to ionizing radiation in sufficient detail. For example, global hypo-acetylation has been reported following ionizing radiation in human cell lines [19, 20] in addition to hypo-methylation of H3K4, but not H3K9, H3K27 or H4K20 [19]. It has been shown that hyper-acetylation of H3K56 occurs at DNA damage foci [21], and an involvement of this mark in DNA repair has been suggested [22]. Further, non-monotonic dose responses to gamma radiation have been reported, exemplified by reduced levels of H3K4me3 after 1h but not after 24h in a lymphoblastoid cell line [19], suggesting dynamic effects of ionizing radiation on histone PTMs which may depend on organism specific factors, dose, and type of radiation.

The zebrafish, with a 70% genetic similarity to humans [23] has become a widely used model organism in radiation studies [7, 24, 25] and environmental epigenetics [26]. The early embryonic development is well described [27] and the early gastrula stage embryo at 50% epiboly (5.5 hpf) produces epigenetic signals with a high signal to background ratio, due to its mainly undifferentiated cell population [28]. Additionally, the histone PTM landscape has been described for this stage [29, 30]. We have recently described effects on the zebrafish embryo transcriptome after 3 h exposure to low dose rates (0.54, 5.4 and 10.9 mGy/h) of gamma radiation [5], as well as the short term and long term effects on the F1 embryo transcriptome after 27 days of parental exposures (8.7 and 53 mGy/h) [31, 32]. These studies also demonstrated adverse reproductive effects and genomic instability in F1 offspring [32]. Furthermore, the genome wide alteration of DNA methylation observed in F1 embryonic offspring of exposed parents indicated a central role of epigenetic mechanisms in response to ionizing radiation [33]. Specifically, the observed transcriptional effects in F1 embryos after parental gamma radiation revealed the involvement of histone modifying genes that could imply a change in chromatin compactness and a potential for epigenetic effects [31]. In this paper, we test the hypothesis that gamma radiation changes chromatin compaction [32], by measuring histone PTM differential enrichment of selected gene loci using exposed zebrafish as the model organism. To investigate whether the mechanisms were specific to zebrafish or conserved between species, effects were compared to Atlantic salmon as an economic and ecologically relevant species.

## Material and methods

### Zebrafish

Zebrafish of the AB wild type strain were obtained from the Norwegian University of Life Sciences (NMBU) zebrafish facility and maintained according to standard operating procedures [5]. The NMBU zebrafish facility is licensed by the Norwegian food inspection authority (NFIA) and accredited by the association for assessment and accreditation of laboratory animal care (AAALAC, license number: 2014/225976). The exposures of fish, including mating and embryo production were approved by NFIA permit number 5793.

### Gamma irradiation

Detailed description of the exposure procedures have been published in [5, 7]. In short, a ^60^Co gamma radiation source with activity of 450 GBq at the NMBU Figaro facility was used for all exposures. Field dosimetry (air kerma rates measured with an ionization chamber) was performed by the Norwegian Secondary Standard Dosimetry Laboratory (Norwegian Radiation Protection Authority, NRPA, Oslo, Norway) [34]. Dose rates to water were estimated as described by Lindbo [35], and used as a proxy for dose rates to the embryos.

### Exposure during zebrafish embryogenesis

One biological sample of 100 zebrafish embryos was exposed to 10.9 mGy/h for 3h, from 2.5 to 5.5 hours post fertilization (hpf), after which the embryos immediately were cross-linked with 1% formaldehyde for 8 min as described in [36]. The cross-linking reaction was quenched with glycine at a final concentration of 125mM after which samples were snap frozen and stored at -80°C until chromatin preparation.

### Exposure during zebrafish gametogenesis

Adult (5 months) zebrafish, 30 males and 30 females, were exposed to 8.7 mGy/h and 53 mGy/h for 27 days [31, 32] and bred 1 year after the end of exposure. The resultant F1 generation was bred at the age of one year to produce F2 embryos. F1 and F2 offspring were collected at the 5.5 hpf stage and 100 embryos sampled for ChIP. Crosslinking was performed as described above and in [36].

Two years after radiation exposure, the parental fish were euthanized with an overdose of Tricaine (MS-222) (Sigma-Aldrich). Gonads were sampled and crosslinked in 1% formaldehyde, fixation quenched with glycine and stored at -80°C until analysis.

### Atlantic salmon embryo exposure

Atlantic salmon eggs and sperm purchased from the Aquagen hatchery Norway were dry *in vitro* fertilized and embryos were kept at 6°C in aerated moderately hard EPA water (pH 6.7) [37]. Exposure containers were positioned at distances representing dose rates of 1, 10, 20 or 30 mGy/h. Fertilized embryos (n = 500) were continuously exposed from one-cell to early gastrula stage (60 day degrees, DD). For each dose rate, 60 DD whole Atlantic salmon embryos from one female were cross linked with 1% formaldehyde for 10 min and fixation quenched with 0,125M final concentration glycine. The cross-linked embryos were washed twice with 20 mL ice cold system water and stored at -80°C until preparation of chromatin. The Atlantic salmon ChIP was performed on F0 generation only due to the long time frame and physical space that would be needed to obtain F1 and F2 generation embryos.

### Chromatin immunoprecipitation of zebrafish

Zebrafish ovaries from control and exposed fish (n = 3) were lysed in 300 μL lysis buffer (1% SDS, 50 mM Tris-HCl, 10 mM EDTA, 1 mM phenylmethylsulfonyl fluoride (PMSF), 20 mM sodium butyrate, 1:100 dilution of protease inhibitor mix (Sigma-Aldrich cat. no. P8340), ground in a mortar and sonicated (Bioruptor pico, Diagenode, Belgium) with 20 cycles before chromatin clearance at 10,000 g for 10 minutes. The chromatin in the supernatant was diluted in urea buffer (2M NaCl, 5M Urea), concentration were determined by Qubit (Thermo-Fisher) and diluted to an equivalent concentration of 10 ng/μL DNA in radioimmunoprecipitation assay (RIPA) buffer (10 mM Tris-HCl pH 7.5, 140 mM NaCl, 1 mM EDTA, 0.5 mM EGTA, 1% (vol/vol) Triton X-100, 0.1% (wt/vol) SDS, 0.1% (wt/vol) Na-deoxycholate, 1 mM PMSF, 20 mM sodium butyrate, 1:100 dilution of protease inhibitor mix). Zebrafish embryo ChIP was carried out as described in [36]. For the exposed embryo samples, two technical replicate ChIPs were performed. In addition, to control for reproducibility, four independent unexposed embryo chromatin samples were prepared and analyzed with duplicate H3K4me3 ChIPs. Antibodies, anti H3K4me3 (cat #. C15410003), anti H3K27me3 (cat # C15410069), anti H3K9me3 (cat #C15410056) and anti H3-pan (cat# C15310135) were purchased from Diagenode, Belgium. Primers targeting upstream sequences of transcription start site (TSS), TSS and gene bodies for the selected genes are listed in S1 Table. The primers were designed in Primer3 v4.0.0 [38, 39] and purchased from ThermoFisher Scientific. The selected genes were: hepatocyte nuclear factor 4 alpha (*hnf4a*, GenBank accession number NM_194368); geminin (*gmnn*, GenBank accession number NM_200086); vascular endothelial growth factor Ab (*vegfab*, GenBank accession number NM_001044855). Precipitated DNA was quantified with qPCR (SYBR green, Roche) using technical duplicates with 2.5 μL ChIP DNA as the input template.

### Chromatin immunoprecipitation of Atlantic salmon embryos

Chromatin was prepared by grinding frozen embryos while adding 2.5 mL E1 buffer (HEPES-KOH, 50 mM, NaCl, 140 mM, EDTA, pH 8.0, 1 mM, Glycerol, 10%, Igepal CA-630, 0.5% Triton-X-100 with additives (protease inhibitor mix, PMSF and Na-butyrate). With a wide P1000 tip, the fluid embryo content, with the chorion left behind, was pipetted to 1.5 mL tubes and centrifuged at 1000g 4°C, 5 min. The supernatant was removed and the pellet was washed in 1 mL E1 and centrifuged at 1000g 4°C, 5 min. Without disrupting the lipid layer, the pellet was redissolved in 200 μL E2 buffer (Tris-HCl, pH 8.0, 10 mM, NaCl 200 mM, EDTA, 1 mM, EGTA 0.5 mM, protease inhibitor mix (1:100 dilution from stock), 1 mM PMSF, 20 mM Na-butyrate), transferred to a clean tube and centrifuged at 1000g, 4°C, 5 min and supernatant removed. Nuclei lysis and chromatin isolation was performed by adding 200 μL lysis buffer (50 mM Tris-Hcl, pH 8.0, 10 mM EDTA, 1% (wt/vol) SDS, protease inhibitor mix (1:100 dilution from stock), 1 mM PMSF, 20 mM Na-butyrate). The lysates were sonicated for 10 cycles and the fragmented chromatin was isolated through centrifugation at 12.000g, 10 min. 4°C. The upper part of the sonicate was transferred to a new tube and the absorbance at 260 nm was determined with a nanodrop device (ThermoFisher scientific). The chromatin was diluted in RIPA buffer to 1 absorbance unit in order to obtain the optimal amount for immunoprecipitation. The immunoprecipitation protocol from this step is similar to that described above for zebrafish. Quantitative analysis of the immunoprecipitated DNA was carried out with real time PCR using 2.5 μl template. Primers were localized over 2 *hnf4a* paralogues at the TSS on chromosome 13 (F: CGGCCGCTAACTTCCATATT, R: ACTCACCATCAGCGAATGGA) and chromosome 15 (F: TGTGGACAAAGACAAGAGGAA, R:AGGCCTCTCTCAATGTTGTCA).

### Statistics used

Statistics were performed in GraphPad Prism v7 (GraphPad Software Inc., San Diego, CA, USA). ANOVA was performed with Tukey post hoc tests on the ovary ChIPs. The precipitation ratios of the exposed and control embryo samples were calculated using the “Remove Baseline” function in GraphPad, and t-tests were performed using the Holm-Sidak method [40] assuming similar standard deviations. P < 0.05 was considered as acceptable significance for differences between experimental and control samples.

## Results

### Gamma radiation exposure of embryogenesis induces histone lysine hyper-methylation

**Fig 1:**
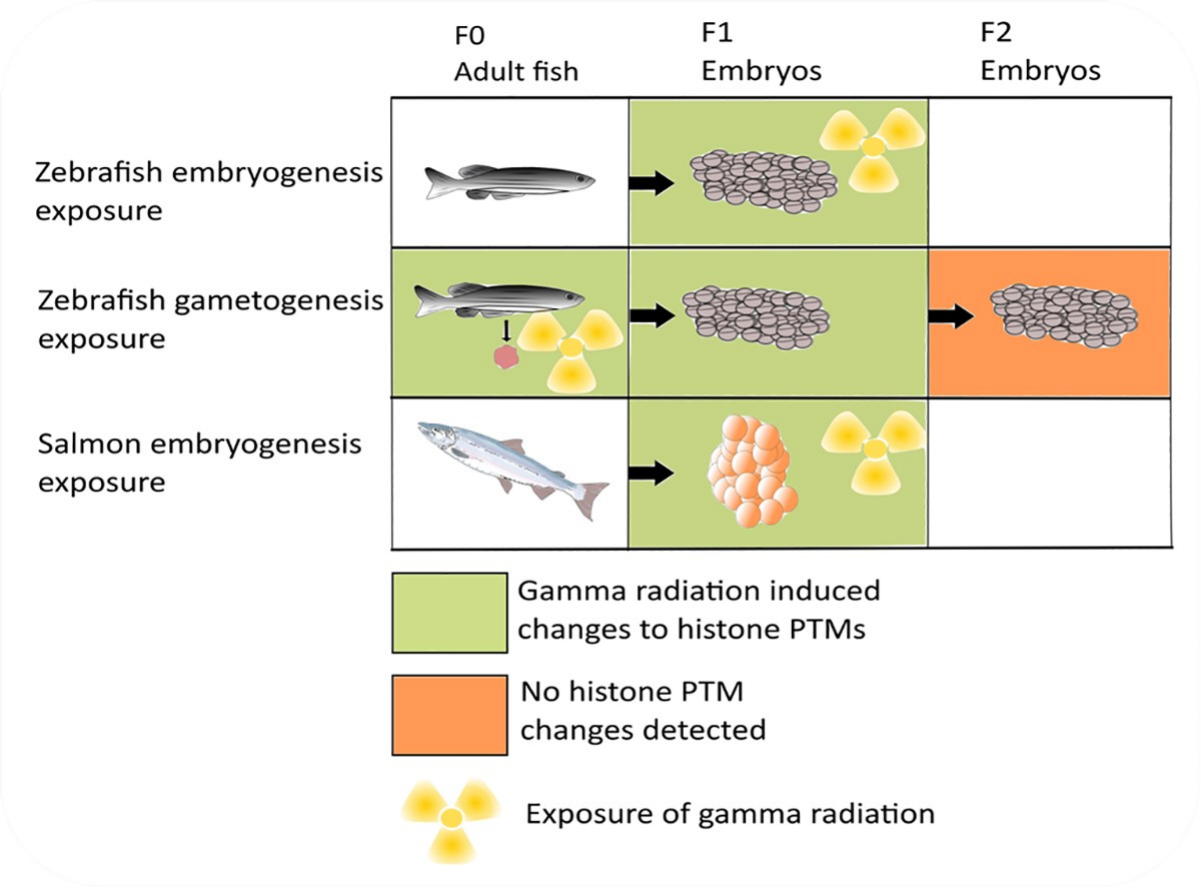
Experimental overview of gamma radiation exposures. Embryos were exposed to gamma radiation (nominal dose rate 10 mGy/h) during blastula to early gastrula stage (5.5 hpf for zebrafish and 60 degree days for Atlantic salmon). Adult zebrafish were exposed for 27 days to affect gametogenesis prior to preduction of F1 and F2 generations. Histone PTM enrichment was measured with chromatin immunoprecipitation and real time PCR changes relative to control is indicated by a green or orange color.

The experimental outline and main results from the series of gamma radiation exposures carried out on zebrafish embryos and adult fish during gametogenesis are presented in Fig 1). Because of long generation cycles for salmon, F1 and F2 offspring were studied in zebrafish only.

**Fig 2:**
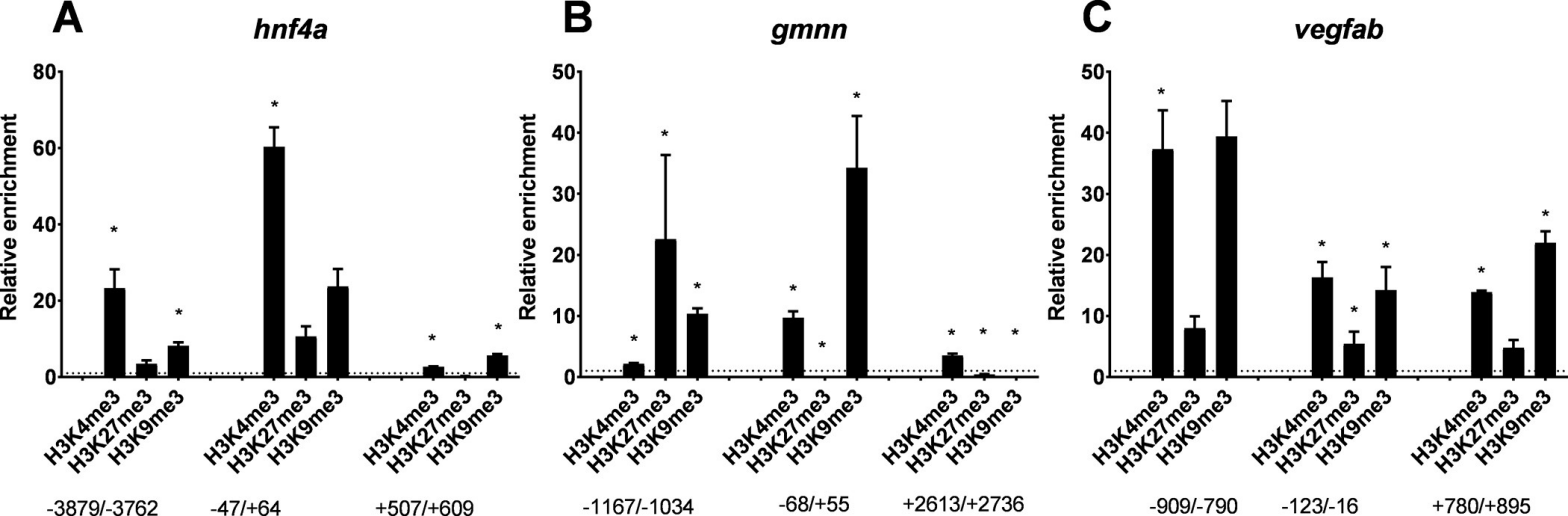
ChIP of directly exposed zebrafish embryos. Fold change enrichment of indicated histone PTMs in directly exposed zebrafish embryos (10.9 mGy/h for 3h) relative to unexposed embryos (5.5 hpf stage). Nucleotide positions are indicated relative to TSS. *p<0.05. Error bars reflect SEM.

Exposure for 3 h to 10.9 mGy/h (total dose 32.7 mGy) gamma radiation during zebrafish embryogenesis, resulted in higher enrichment of H3K4me3, H3K27me3 and H3K9me3 at 5.5 hpf relative to non-irradiated controls, for upstream sequences, TSS as well as the gene body of the analyzed genes (Fig 2A–2C). At the transcriptionally upregulated gene (*hnf4a* log2FC = 0.8 [7], Fig 2A), an increase of H3K4me3 was observed at the TSS. Interestingly, the TSS of the downregulated genes (*vegfab* log2FC *= -0.7; gmnn log2FC = -0.3 [7]*; Fig 2B and 2C) was enriched in H3K4me3 compared to the unexposed control while the repressive mark, trimethylated K9, showed significant enrichment as well (Fig 2A–2C).

### Exposure of adult fish induces reduction of histone H3 and H3K4me3 in zebrafish ovaries

**Fig 3:**
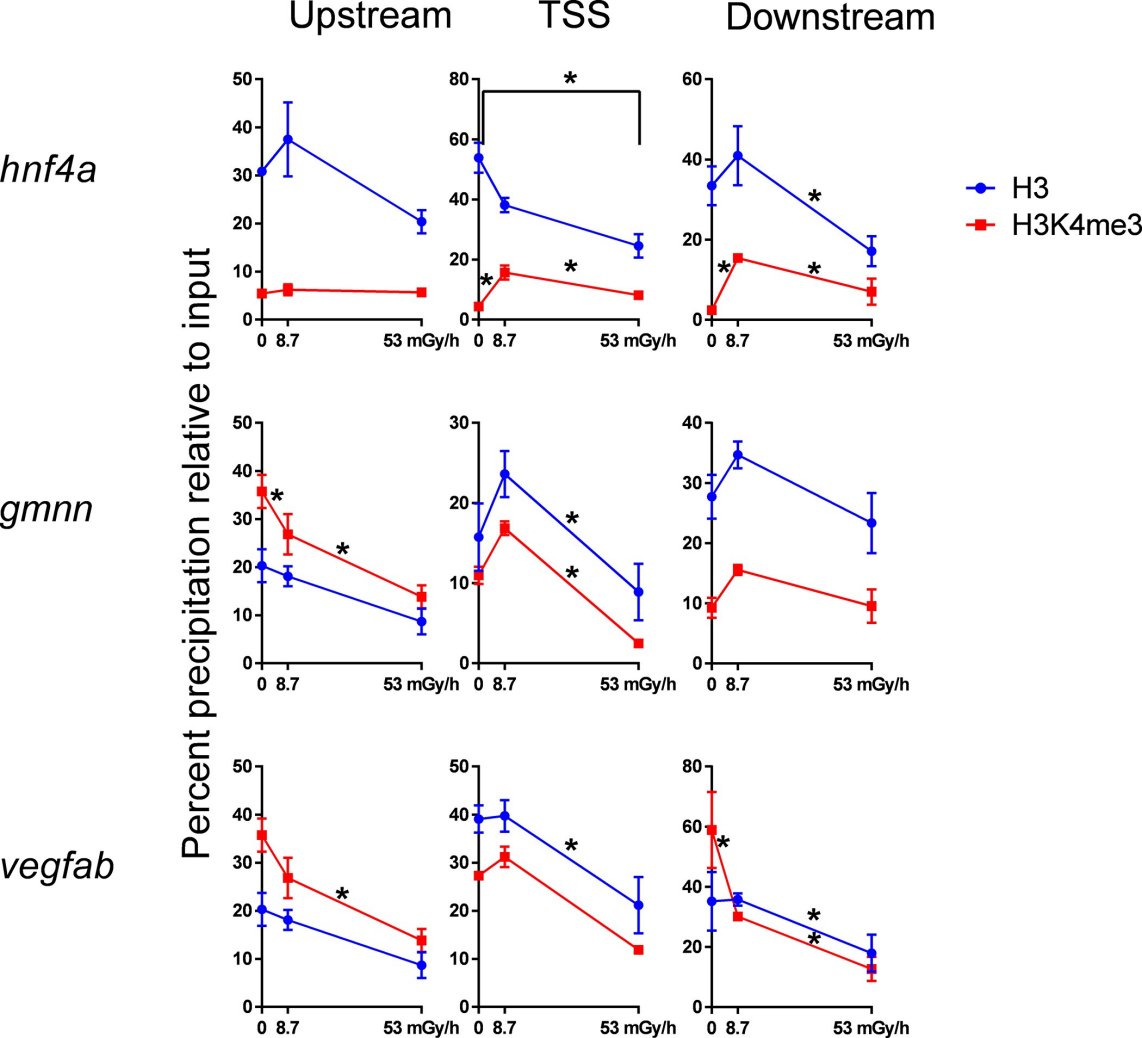
ChIP of ovaries from exposed parental generation. Enrichment of H3 and H3K4me3 in zebrafish ovaries after 27 day exposure during gametogenesis (control, 8.7 and 53 mGy/h). Enrichment is measured upstream of TSS, at TSS and downstream of TSS (coordinates given in Fig 1). *p<0.05. Error bars reflect SEM.

Two years post irradiation, the ovaries of exposed adult fish showed H3 enrichment tended to be correlated with H3K4me3 enrichment, with the lowest enrichment occuring at the highest dose rate (53 mGy/h, total dose 34 Gy, Fig 3). On *gmnn* and *vegfab* loci, *a* significant decline in H3 enrichment was observed only at the highest dose rate, while a dose response was seen at the *hnf4a* loci.

### Exposure of adult fish during gametogenesis reveals histone hyper-methylation in F1 but not F2 embryos

**Fig 4:**
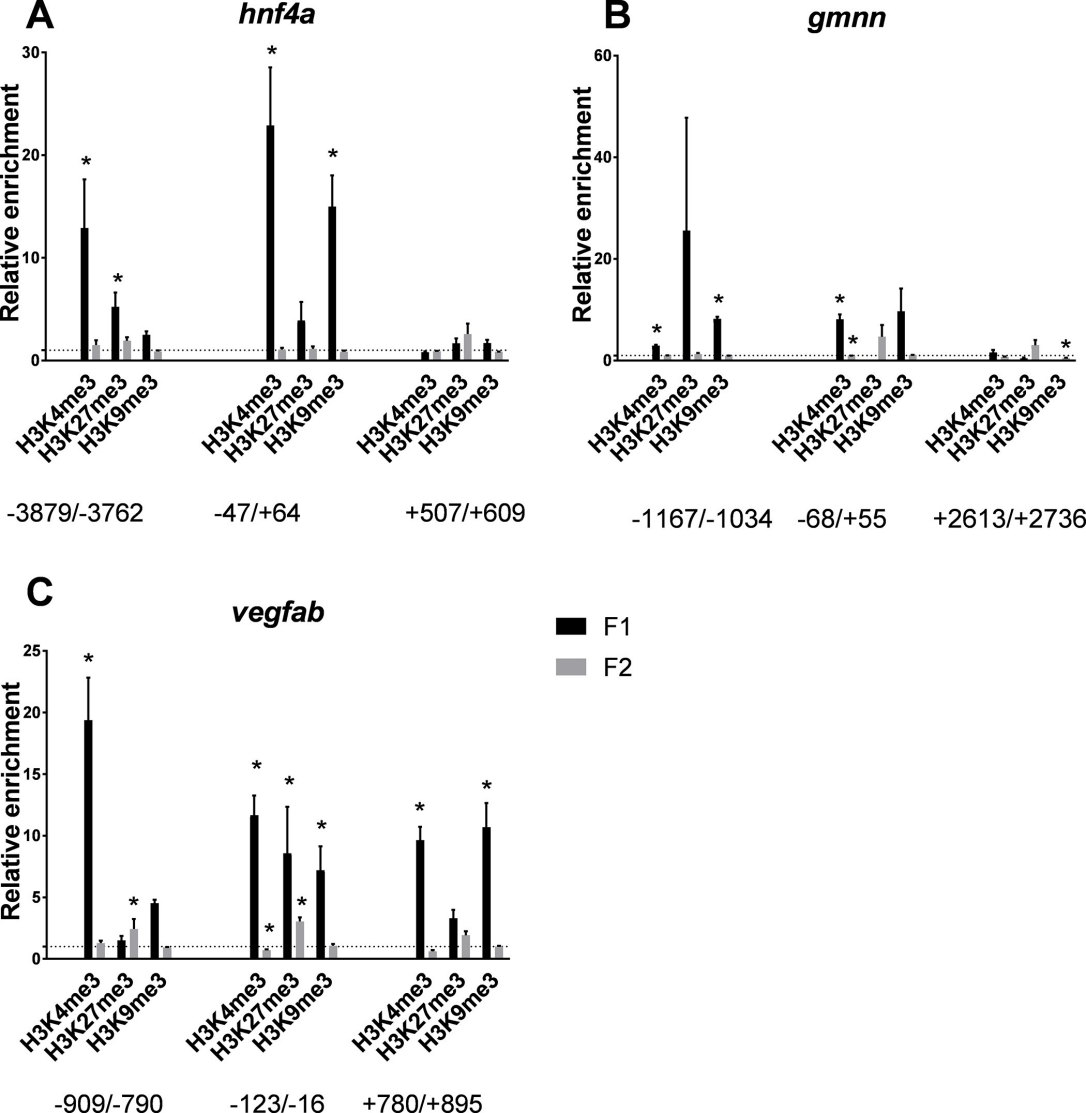
ChIP analysis of F1 and F2 offspring of exposed parents. Fold change enrichment (relative to controls) of indicated histone PTMs in F1 and F2 zebrafish embryos (5.5 hpf) after parental gamma radiation exposure. Nucleotide positions indicated relative to TSS. *p<0.05. Error bars reflect SEM.

Gamma radiation exposure throughout gametogenesis (8,7 mGy/h, total dose 5.6 Gy) revealed hyper-methylated histone residues at the three selected loci in first filial embryos bred one year after exposure of the parents (Fig 4A–4C).

The F1 transcription levels for the genes showed similarities to the directly exposed embryos with *hnf4a* (log2FC = 1.56) being upregulated, *gmnn* (log2FC = -1.31) downregulated, but *vegfab* was not differentially expressed in F1 offspring after the gametogenesis exposure [32]. On the *hnf4a* locus, H3K4me3 and H3K27me3 were significantly enriched upstream of TSS, while H3K4me3 and H3K9me3 were enriched at TSS (Fig 4A). For *gmnn* (Fig 4B) and *vegfab* (Fig 4C) higher enrichment of H3K4me3, H3K27me3 and H3K9me3 compared to controls was seen. When investigating the F2 generation, no or very small difference in enrichment between the embryos originated from the exposed fish or control fish of the same age were observed on the analyzed genes (Fig 4A–4C).

### Gamma radiation exposure of Atlantic salmon embryos reveals conserved effects on H3K4me3 enrichment

**Fig 5:**
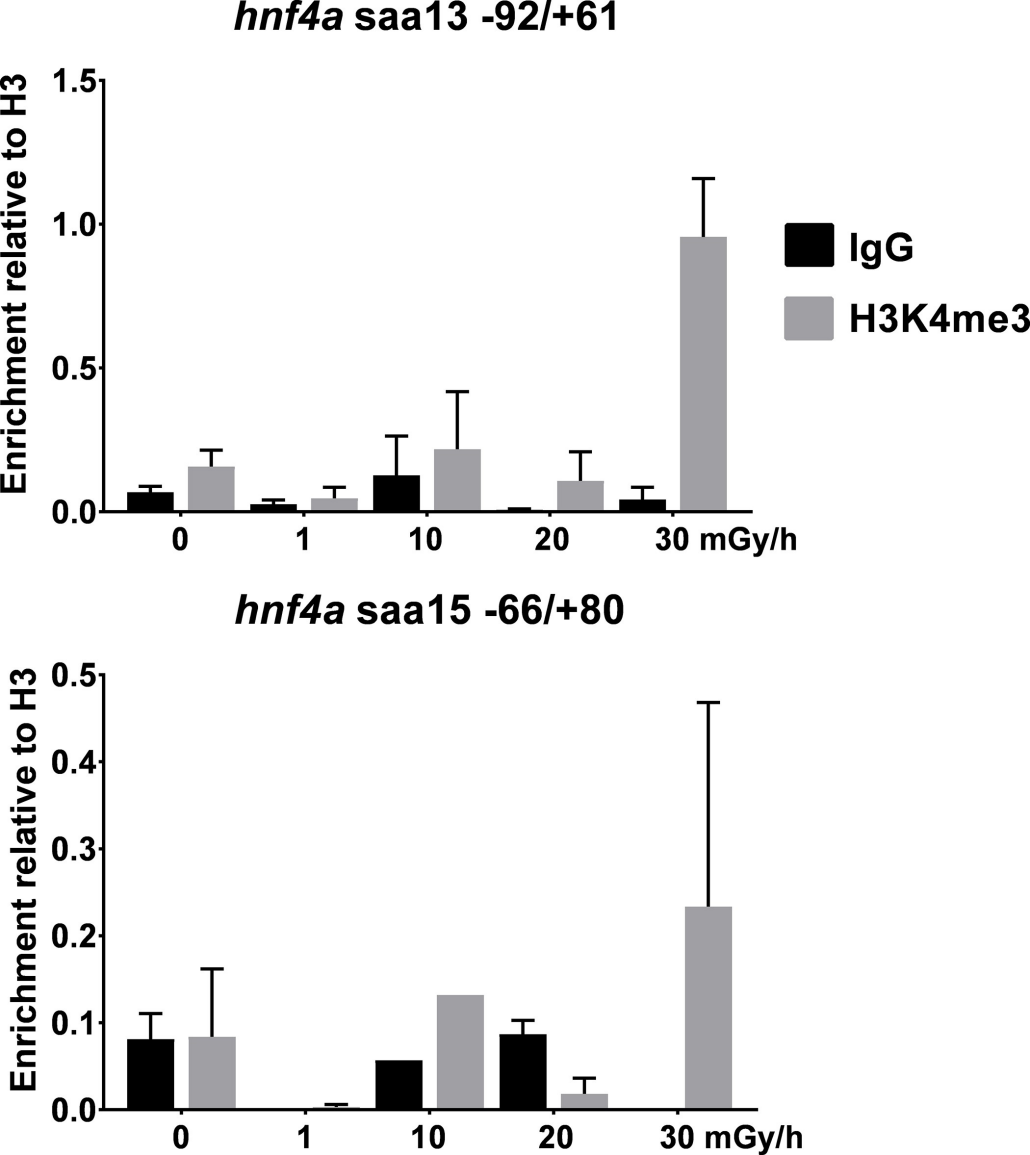
Atlantic salmon embryo ChIP. Atlantic salmon embryo (60 degree days; 50% epiboly) ChIP analysis enrichment at TSS of IgG and H3K4me3 after exposures for 10 days at 0–30 mGy/h. Error bars reflect the SEM of two technical replicates on one biological sample made from pools of 60 embryos.

Atlantic salmon was exposed to 0, 1, 10, 20 and 30 mGy/h for 10 days at 6°C (60 degree days stage) corresponding to the early gastrula stage at 5.5 hpf for zebrafish. For Atlantic salmon a pronounced enrichment of H3K4me3 was induced by the dose rate 30 mGy/h gamma radiation at the TSS sequences of the *hnf4a* loci upstream sequences, significantly at the paralog gene on chromosome 13, with a similar trend at chromosome 15 locus though with high deviation in the results (Fig 5). No significant changes were seen at the lower dose rates.

## Discussion

Zebrafish exposure to gamma radiation causes damages in cells and organs such as changes in gene expression, increased DNA damage, RNS/ROS production and developmental phenotypic aberrations at the organismal level, in addition to defective phenotypes in offspring [5, 7, 31, 32]. To address how chromatin structure relates to gamma radiation effects, a series of experiments were performed where histone PTM levels were measured at a set of selected target loci. The zebrafish early gastrula stage embryo (5.5 hpf) is well suited for epigenetic studies because of the still relatively multipotent state of cells, producing a high signal to noise ratio when performing whole embryo epigenetic analysis. We demonstrated that gamma radiation induced changes on histone lysine methylation enrichment on three zebrafish genes localized on different chromosomes (*hfn4a*, chr. 23; *gmnn*, chr. 19; *vegfab*, chr. 4). These changes were seen after direct exposure of zebrafish embryos (10.9 mGy/h), in ovaries 2 years after exposure of adult zebrafish (8.7 and 53 mGy/h) and in F1 embryos bred one year after exposure of the parents (8.7 mGy/h) (Fig 1). In a distant related teleost species, Atlantic salmon, a similar effect is observed at the *hnf4a* gene after exposure of embryos, albeit at a higher dose rate (30 mGy/h) than the direct effects seen in zebrafish embryos. The *Hnf4a* protein family is important for genetic control of embryonic development [41] and aberrant regulation is related to endocrine disruption (diabetes) and cancer development [42]. Based on our work on gamma radiation exposed zebrafish, we hypothesized that gamma radiation would induce change(s) in chromatin structure. Indeed, zebrafish embryos exposed to 10.9 mGy/h gamma radiation for 3 hours, reveal enriched levels of H3K4me3, H3K27me3 and H3K9me3 at all genes investigated compared to control (Fig 2, suggesting that these histone PTMs could be potential biomarkers for ionizing radiation. However, a notable observation is a disagreement between gamma radiation induced enrichment of permissive and repressive histone PTM marks at TSS and upstream sequences in directly exposed embryos compared to the observed [7] and predicted differential gene expression pattern. Irrespective of whether the transcription is upregulated or downregulated [7], hyper-enrichment of H3K4me3 compared to controls is seen at TSS and upstream sequences. In general, higher enrichment of activating marks are associated with enhanced transcriptional activity [43]. The observed disagreement between differential gene expression and histone PTM pattern may be explained by a shut-down of the transcriptional machinery as a protection mechanism, wherein new RNA synthesis is repressed until the genome is repaired after DNA damage [44]. Increased amounts of H3K27me3 and H3K9me3 after irradiation suggests polycomb group (PcG) proteins induced heterochromatinization as a mechanism for genome repression [45]. Further, gamma radiation has been reported to induce a long non-coding RNA, PARTICLE, shown to interact with both H3K9 methylase G9a and polycomb group component SUZ12, mediating H3K27 methylation [46].

To make the results relevant to not only oviparous species, we performed a transgenerational experiment with parental fish exposed through gametogenesis for 27 days. Significant effects at the level of chromatin were found in ovaries from the parental zebrafish exposed to radiation during gametogenesis (Fig 3). At the upstream sequences and in the gene body, the H3K4me3 enrichment change was associated with decline in histone H3 levels. This may reflect a nucleosome density with a total chromatin collapse at the 53 mGy/h dose rate, and corresponds to ovary deformities and reproductive loss reported by [5]. Different dynamics of the enrichment patterns suggest loci and histone PTM dependent non-monotonic dose responses. This can be exemplified by the significantly higher enrichment of H3 at *hnf4a* TSS at 8.7 mGy/h compared with a reduction in enrichment at 53 mGy/h (Fig 3). The deviant histone PTM patterns could possibly be carried through to the F1 directly via gametes, co-exposed during the gametogenesis. Epigenetic memory is postulated to be transferred through generations mediated by DNA-methylation, non-coding RNAs or modified histones [47]. In several species, transgenerational effects have been detected in many generations after the parents were exposed to ionizing radiation, reviewed in [47]. For example, transgenerational memory has been shown to last for 14 generations in *Caenorhabditis elegans*, with histone modifications (H3K9me3) as a possible epigenetic information carrier [48]. In light of this, the hyper-enrichment of H3K9me3 in F1 embryos (Fig 4) may be explained by either a co-exposure direct effect or by an epigenetic multigenerational memory transferred from the exposed parents. In the F2 generation after gametogenesis exposure (Fig 4), there were negligible differences at the studied loci, suggesting that the changes in histone modifications on the investigated targets are not retained in a multi-transgenerational manner. This does not exclude the possibility that other loci and or histone PTMs could show transgenerational effects after gamma irradiation.

To elucidate wether the findings are specific to zebrafish, or if there is a conserved mechanism relevant to other teleost fish species, we set up a similar study using 60 degree days Atlantic salmon early gastrula stage embryos [49], which is a comparable developmental stage to zebrafish 5.5 hpf embryos. Indeed, H3K4me3 hyper-enrichment, as observed in zebrafish, was detected in Atlantic salmon embryos on the TSS of *hnf4a* locus, although at a higher dose rate (Fig 5). A second whole genome duplication in addition to the common teleost genome duplication [50, 51], results in two hnf4a gene paralogs in Atlantic salmon and the similar results are seen on each chromosome. However, this suggests that the observed effect/mechanism may be conserved between zebrafish and Atlantic salmon and possibly other species. Concern has been raised about the quantitative aspect of the ChIP assay, and several suggestions have been made how to make the ChIP assay for quantitative assessment [52]. However, in our experimental set up with zebrafish embryos we showed reproducible H3K4me3 enrichments from independently prepared chromatin samples (S1 Fig). It is not known whether the observed gamma radiation induced histone lysine hyper-methylation is associated with the reported phenotypic effects and changes in gene transcription [5, 7, 31, 32], or whether it is a transient biological response to protect the DNA and the embryo. One hypothetical effect of epigenetic inheritance is to prepare the next generation for the expected environmental conditions. Another model to consider is whether the enrichment of histone PTMs is a similar toxicological response to that described for carcinogenic metals [53, 54] or a response to cellular oxidative stress [55]. The finding of elevated ROS in directly exposed embryos and oxidative stress in offspring from exposed parents [5, 7], could in principle induce chromatin modifications. One additional possibility that could in principle result in elevated cross-linking in exposed samples is radiation induced cross-linking [1]. However, this is not compatible with the development of exposed embryos up to 5 days post fertilization. The present study demonstrates that chromatin organisation is affected by direct exposure of gamma radiation. In addition, we show that the induced changes are passed on to the subsequent generation in zebrafish. From the results of a parallel study with Atlantic salmon embryos kept in a similar exposure scenario it was concluded that the observed enrichment of H3K4me3 at same locus is conserved between the two evolutionary distant species. The chromatin responds to radiation with different mechanisms in ovaries compared to embryos, for example while radiation induced higher H3K4me3 levels in embryos, it lead to decreased H3K4me3 levels in adult ovaries. Further insight into how the epigenome responds to gamma radiation might be gained by applying genome-wide techniques such as ChIP sequencing, chromatin accessibility and chromatin conformation capture assays.

## Supporting information

S1 Fig Reproducibility of ChIP assay. H3K4me3 enrichment on four independent chromatin preparations of unexposed embryos. The loci are localized upstream of the transcriptional start site. The unspecific binding (empty beads) were negligible on all loci. Each enrichment profile is represented as percent of precipitation of input and error bars reflects SEM of two technical replicates.
(TIF)

S1 Table Primer sequences and amplicon placement relative to TSS in the given gene loci. (DOCX)

## Abbreviations

ChIP: Chromatin immunoprecipitation
hpf: Hours post fertilization
mpf: Months post fertilization
PTM: Post translational modification
ROS: Reactive oxygen species
RNS: Reactive nitrogen species
