# Supplementary figures and images for: Gamma radiation induces locus specific changes to histone modification enrichment in zebrafish and Atlantic salmon
Source: PLoS One. 2019 Feb 13;14(2):e0212123. doi: 10.1371/journal.pone.0212123 (PMC6373941; doi:10.1371/journal.pone.0212123)

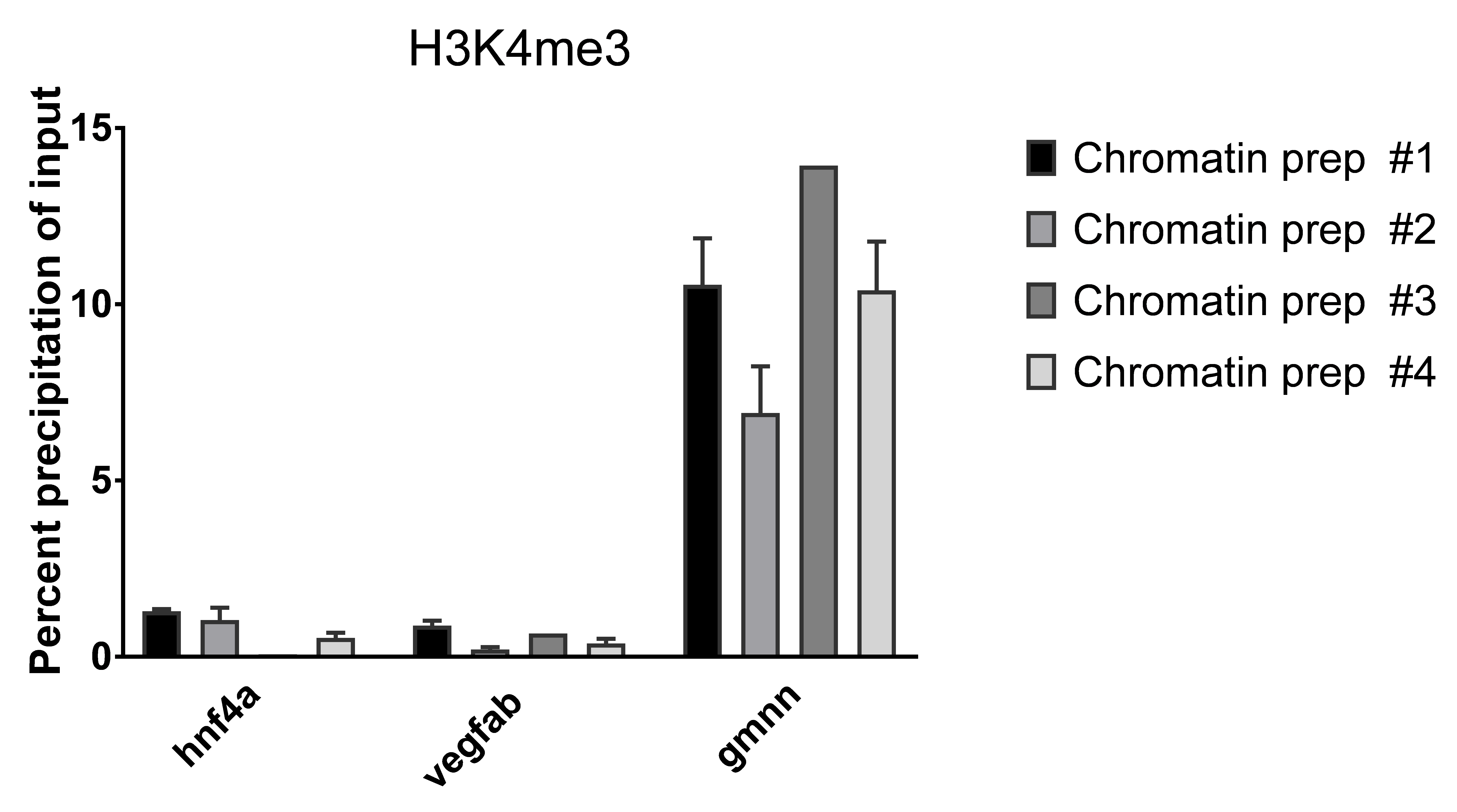

Supplement: S1 Fig — H3K4me3 enrichment on four independent chromatin preparations of unexposed embryos. The loci are localized upstream of the transcriptional start site. The unspecific binding (empty beads) were negligible on all loci. Each enrichment profile is represented as percent of precipitation of input and error bars reflects SEM of two technical replicates. (TIF) [file pone.0212123.s001.tif]
