# Supplementary material for: Gamma radiation induces locus specific changes to histone modification enrichment in zebrafish and Atlantic salmon
Source: PLoS One. 2019 Feb 13;14(2):e0212123. doi: 10.1371/journal.pone.0212123 (PMC6373941; doi:10.1371/journal.pone.0212123)
Supplement: S1 Table — (DOCX) [file pone.0212123.s002.docx]

| **Gene** | **Start pos. rel. to TSS** | **End position rel. to TSS** | **Forward primer** | **Reverse primer** | **Annealing temp. (°C)** |
| --- | --- | --- | --- | --- | --- |
| *hnf4a* | -3879 | -3762 | tttcagaggctcaggtcagg | cataagtatcagggccagcg | 60 |
| *hnf4a* | -47 | +64 | caatcacagcgagcctcatc | tctagagcgtttggacaccc | 60 |
| *hnf4a* | +507 | +609 | tcatacagctggcttgggat | gaggattggctgcgaaacat | 60 |
| *hnf4a* | +1715 | +1825 | catgaatgctgccaaccact | agaaccccttgcatccatca | 60 |
| *cebpa* | -4332 | -4200 | tctctggcgcaacttccaat | tctgggtcgaacaaatgggt | 60 |
| *cebpa* | -1818 | -1679 | cgcttgggagtattgcgaaa | acagagatgtgcagcctgat | 60 |
| *cebpa* | -123 | +21 | taggtctatcagtgcgtccg | acttgcaacctcagtgtgtg | 60 |
| *cebpa* | +498 | +603 | gatgtatggctgcctgaacg | tcccgaggctcttgtttgat | 60 |
| *cebpa* | +1840 | +1943 | gcagtgaagtcctgtcttgc | cactgaaaccatctctgctcg | 60 |
| *vegfab* | -909 | -790 | atgaccgagaagccccaaaa | tgagcagtctacgtgcagaa | 60 |
| *vegfab* | -123 | -16 | acttaactgagtccacgctga | tcagctgtcggtctgttctt | 60 |
| *vegfab* | +780 | +895 | tgaatgtaatgtgcctgcgc | gtggtcagatatgctcgttca | 60 |
| *gmnn* | -1167 | -1034 | tctacagtcgtcacacaccc | tcaccctttccttccagcat | 60 |
| *gmnn* | -68 | +55 | gtctcagtacgcgcgaattc | acaatcgctcgcttcacaaa | 60 |
| *gmnn* | +2613 | +2736 | aaaaggaagatgtggagcgc | ataggcctcttgtgtgaccc | 60 |
